# Improving initial infectivity of the *Turnip mosaic virus* (TuMV) infectious clone by an mini binary vector via agro-infiltration

**DOI:** 10.1186/1999-3110-54-22

**Published:** 2013-08-28

**Authors:** Yen-Yu Lin, Meng-Mei Fang, Pin-Chun Lin, Ming-Tzu Chiu, Li-Yu Liu, Chan-Pin Lin, Shih-Shun Lin

**Affiliations:** 1 Institute of Biotechnology, College of Bioresources and Agriculture, National Taiwan University, 81, Chang-Xing ST.,, Taipei, 106 Taiwan; 2 Genome and Systems Biology Degree Program, National Taiwan University, 1, Sec. 4, Roosevelt Rd.,, Taipei, 106 Taiwan; 3 Agriculture Biotechnology Research Center, Academia Sinica, 128, Sec. 2, Academia Rd.,, Taipei, 115 Taiwan; 4 Department of Plant Pathology and Microbiology, College of Bioresources and Agriculture, National Taiwan University, 1, Sec. 4, Roosevelt Rd.,, Taipei, 106 Taiwan; 5 Department of Agronomy, National Taiwan University, 1, Sec. 4, Roosevelt Rd.,, Taipei, 106 Taiwan

**Keywords:** *Turnip mosaic virus*, Infectious clone, Initial infectivity, Mini binary vector

## Abstract

**Background:**

The *in vivo* infectious clone of *Turnip mosaic virus* (TuMV), p35S-TuMV, was used on plant pathology research for many years. To activate p35S-TuMV, the plasmid was mechanically introduced to the local lesion host *Chenopodium quinoa*. However, low infectivity occurred when the TuMV from *C. quinoa* was transferred to the systemic host *Nicotiana benthamiana*.

**Results:**

To increase the efficiency of initial infectivity on *N. benthamiana*, the expression of the TuMV infectious clone by a binary vector that directly activates viral RNA through agro-infiltration is considered to be a good alternative. The size of the binary vector by agro-infiltration is usually large and its backbone has numerous restriction sites that increase difficulty for construction. In this study, we attempted to construct a mini binary vector (pBD003) with less restriction sites. The full-length cDNA of TuMV genome, with or without green fluorescence protein, was inserted in pBD003 to generate pBD-TuMV constructs, which were then individually introduced to *N. benthamiana* plants by agro-infiltration. Symptom development and ELISA positivity with TuMV antiserum indicated that the pBD-TuMV constructs are infectious. Moreover, the initial infectivity of a mild strain TuMV-GK, which contains an R_182_K mutation on HC-Pro, constructed in the pBD003 vector was significantly increased by agro-infiltration.

**Conclusion:**

Thus, we concluded that the newly constructed mini binary vector provides a more feasible tool for TuMV researches in areas, such as creating a mild strain for cross-protection, or a viral vector for foreign gene expression. In addition, the multiple cloning sites will be further cloned in pBD003 for convenience in constructing other viral infectious clones.

**Electronic supplementary material:**

The online version of this article (doi:10.1186/1999-3110-54-22) contains supplementary material, which is available to authorized users.

## Background

The infectious clone of the RNA virus is a plasmid containing the full-length cDNA form of the viral genome. Under a suitable promoter, the DNA form of a viral genome can be transcribed into an initial infectious viral RNA by *in vitro* or *in vivo* methods to enable research into molecular virology. The viral infectious clone was developed in the late 1970s, and benefited by the recombinant DNA and reverse-transcription techniques (Boyer and Haenni, 1994). Currently, in an infectious clone, it is possible to introduce a mutation on the viral genome at a particular position, or to process recombination between two strains of a particular virus. The infectious clone has become an indispensable tool in the research of plant and animal viruses (Kobayashi et al., 2007; Schnell et al., 1994).

Initial infectivity refers to the triggering of transcription of the DNA form of a viral genome to generate infectious viral RNA, which begins to establish the life phase of a virus. Thereafter, the viral RNA can self-replicate and has the ability to subsequent infect the host plant. Based on differences in initial processing, infectious clones are classified as either *in vitro* or *in vivo*. The *in vitro* infectious clones require phage promoters, such as *T7*, *T3*, or *Sp6* promoter to generate viral RNA by *in vitro* transcription. In contrast, the CaMV *35S* promoter is usually used with the *in vivo* infectious clone and the promoter is recognized by host RNA polymerase II (pol II) in the plant nucleus to transcribe viral RNA *in vivo*. (Angenent et al., 1989; Heaton et al., 1989; Janda et al., 1987; Lin et al., 2002; Melton et al., 1984). Once the viral RNA is transcribed from DNA, the virus life cycle is initiated, and consequent infectivity is established.

The initial infectivity rate may be affected by RNase contamination, inoculation method, host characteristics, and viral activity. The RNase contamination specifically indicated that the operation with the *in vitro* transcript that was exposed to an RNase-rich environment might damage the viral RNA during the inoculation. In a previous study, plants of *Chenopodium quinoa* were inoculated with a DNA form of an *in vivo* infectious clone of *Zucchini yellow mosaic virus* (ZYMV) by mechanical inoculation for local lesion formation, and the generated virus was then transferred from a *C. quinoa* plant to a zucchini squash plant (Lin et al., 2002). It has been reported that the host characteristics of squash cannot cause initial infectivity by direct mechanical inoculation with the *in vivo* infectious clone of ZYMV (Lin et al., 2002). The *C. quinoa* plants can be directly inoculated with the DNA of an *in vivo* infectious clone, with high efficiency of the infectivity and without the risk of RNase contamination. This process is easy and less costly than *in vitro* transcription. However, the secondary inoculation for the transfer of a virus from *C. quinoa* to a systemic host plant is somehow difficult especially for a viral mutant, which does not form distinct local lesions. The *Turnip mosaic virus* (TuMV) has low infectivity on *Nicotiana benthamiana* plants when the inoculum is prepared from infected *C. quinoa* tissue (unpublished data). It appears that the TuMV activity was affected by an unknown-mechanism when the virus existed in *C. quinoa*. In addition, the inefficiency of the initial infectivity of TuMV (less than a 10% success rate) in *N. benthamiana* plants caused by particle bombardment with an *in vivo* TuMV infectious clone was also a problem (unpublished data). These difficulties generate a limitation for studying the functions of TuMV mutants, in which weaker infectivity may be incurred by mutations. In addition, similar to ZYMV, the DNA of the *in vivo* infectious clone of TuMV also does not efficiently cause initial infectivity on the systemic host *N. benthamiana* by mechanical inoculations.

To solve this problem, we attempted to transfer the *in vivo* infectious clone into the nucleus of a host plant by agro-infiltration, thus bypassing the need for *C. quinoa* inoculation. The binary vector was designed for *Agrobacterium tumefaciens* to transfer the T-DNA into a plant cell nucleus (Joh and VanderGheynst, 2006). To date several *in vivo* infectious clones have been designed to deliver by the binary vector through agro-infiltration, such as *Citrus yellow mosaic virus* (CYMV), *Tobacco rattle virus* (TRV), and *Begomovirus*, to increase the initial infectivity rate in *N. benthamiana* (Cui et al., 2005; Huang and Hartung, 2001; Liu et al., 2002). Agro-infiltration provides a good strategy to overcome the lower rate of initial infectivity. However, the size of the binary vector is usually large (> 10 kb) and difficulties may be encountered in ligation with large potyviral genome inserts, which are usually approximately 10 kb in length. In addition, numerous commonly used restriction enzyme sites exist on the backbone of the binary vector, limiting the construction strategy with regard to the use of restriction sites.

In this study, we constructed a mini binary vector (4 kb), pBD003, in which most of restriction enzyme sites on the plasmid backbone were removed. The full length of TuMV cDNA was constructed in pBD003, downstream from the CaMV *35S* promoter, with suitable restriction enzyme digestion and ligation to generate an *in vivo* pBD-TuMV infectious clone. We further generated pBD-TuMV-GFP, which carries a *green fluorescent protein* (*GFP*) gene for tracking the virus in host. In addition, a TuMV mild strain, TuMV-GK, which has R_182_K mutation on HC-Pro, was also constructed in pBD003 vector in this study. The biological activities of the pBD-TuMV series were analyzed on the local lesion host *C. quinoa* by mechanical inoculation and on the systemic host *N. benthamiana* by agro-infiltration. The initial infectivity of these pBD-TuMV series was detected and confirmed by indirect enzyme-linked immunosorbent assay (ELISA), which verified the infectivity of these infectious clones. Thus, these pBD-TuMV series of infectious clones provide a powerful tool for further research into topics, such as creating a mild strain for cross-protection. Multiple cloning sites (MCS) will be introduced in pBD003 in the future, and will be suitable for other viral infectious clone construction.

## Methods

### Plant material and growth condition

Plant-related experiments, such as agro-infiltration and viral inoculation were performed in a greenhouse (16 h light/8 h darkness, 20°C to 25°C). The seeds of *C. quinoa* and *N. benthamiana* were sown in peat soil. After germination, seedlings were transferred to soil and fertilized with Peters 20-20-20 once a week.

### Construction of a mini binary vector

**Figure 1:**
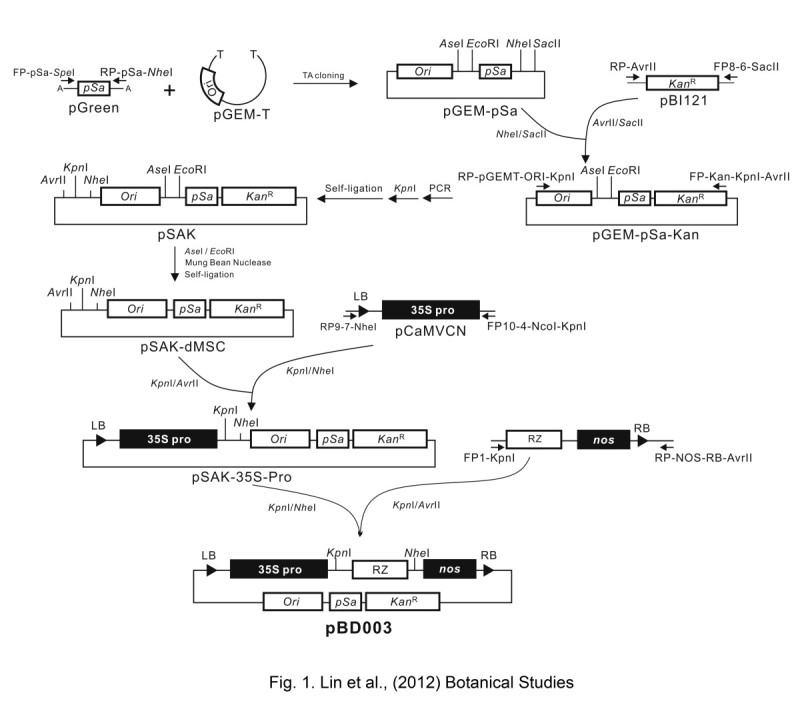
**Construction of the pBD003 binary vector.** Construction procedure of the mini binary vector - pBD003 contains an agrobacterium replication origin (*pSa*) and an *E. coli*. replication origin (*Ori*) for the plasmid replication in agrobacterium and *E. coli*, respectively*.* The *Cauliflower mosaic virus* (CaMV) *35S* promoter (*35S*), the ribozyme (*RZ*) sequence, and the *nopaline synthase* terminator (*nos*) were constructed and inserted between the left border (LB) and right border (RB) on pBD003. The restriction sites frequently used in cloning on the vector backbone were mutated by PCR-mediated mutagenesis. The *Kpn* I restriction site was created between the CaMV *35S* promoter and ribozyme sequence for further cloning or construction.

The strategy for constructing a mini binary vector with the *35S* promoter and *nos* terminator is summarized in Figure 1. The *pSa* origin was amplified by polymerase chain reaction (PCR) with the primer pair FP-pSa-SpeI (5′- TCTTATCACTAGT AAGCCCGAGAGGTTGCCGCC -3′) and RP-pSa-NheI (5′- GGAGGGTAGGCTAGC GTTATCCACGTGAAACCGC -3′), which contain *Spe* I and *Nhe* I (underlined), respectively, from pGreen (Hellens et al., 2000) and was cloned into the pGEM-T vector (Promga) to generate pGEM-pSa. Next, the *kanamycin* resistance gene (*Kan*^R^) was amplified from pBI121 binary vector with the primer pair FP-8-6-SacII (5′- GCCTGTGATCATCCGCGG TTTCAAAATCGGCTCCG -3′) and RP-AvrII (5′- GGTTTTTTTGTTTGCAAGCCTAGG CAGATTACGCGC -3′), which contains *Sac* II and *Avr* II (underlined), respectively. The PCR fragment of *Kan*^R^ gene was digested with *Avr* II/*Sac* II and ligated to *Nhe* I/*Sac* II-digested pGEM-pSa vector to generate pGEM-pSa-Kan. To remove the unnecessary DNA backbone, the primer pair FP-Kan-KpnI-AvrII (5′- GCTCCCGGTACC GCCAGGCGGCCTAGG TTTCAAAATCGG -3′) containing *Kpn* I and *Avr* II (underlined), and RP-pGEMT-ORI-KpnI (5′- GCCTCACTGATTAAGCATTGGTACC TGTCAGACC -3′) containing *Kpn* I, (underlined) was used to amplify the 2,916 bp fragment of pGEM-pSa-Kan that included the *Ori*-*pSa*-*Kan*^*R*^ sequence by PCR. The PCR product was digested with *Kpn* I and self-ligated to generate pSAK. Thereafter, the pSAK plasmid was digested with *Ase* I/*Eoc* RI to remove the restriction enzyme sites and the digested product was treated with Mung Bean nuclease to produce blunt ends and then self-ligated to generate pSAK-dMSC. The left border (LB) and CaMV *35S* promoter sequence were amplified from pCaMVCN (Pharmacia) with the primers FP10-4-NcoI-KpnI (5′- CCGGCCGCCATGGGGTACC CCTCTCCAAATGAAAT -3′) and RP9-7-NheI (5′- GCGATGATCACAGGCTAGC AACGCTCTGTCATCG -3′), which contain *Kpn* I/*Nco* I, and *Nhe* I (underlined), respectively. The PCR product was then digested with *Kpn* I/*Nhe* I and ligated with *Kpn* I/*Avr* II-digested pSAK-dMSC vector to generate pSAK-35S-Pro. Finally, the DNA region including the ribozyme, *nos*, and right border (RB) was amplified from pTRV2 plasmid (Liu et al., 2002) with the primers FP1-KpnI (5′- ACTTGGTACC GTCTGTACTTATATCAGTACACTGACGAG -3′) and RP-NOS-RB-AvrII (5′- TTGGCACCTAGG TACAAATGGACGAACGGATAAAC -3′), which contain *Kpn* I, and *Avr* II (underlined), respectively. The PCR product was digested with *Kpn* I/*Avr* II and ligated with the *Kpn* I/*Nhe* I-digested pSAK-35S-Pro vector to generate the mini binary vector pBD003 of 4,141 bp.

### Construction of *in vivo* full-length cDNA clones of TuMV for agro-infiltration

**Figure 2:**
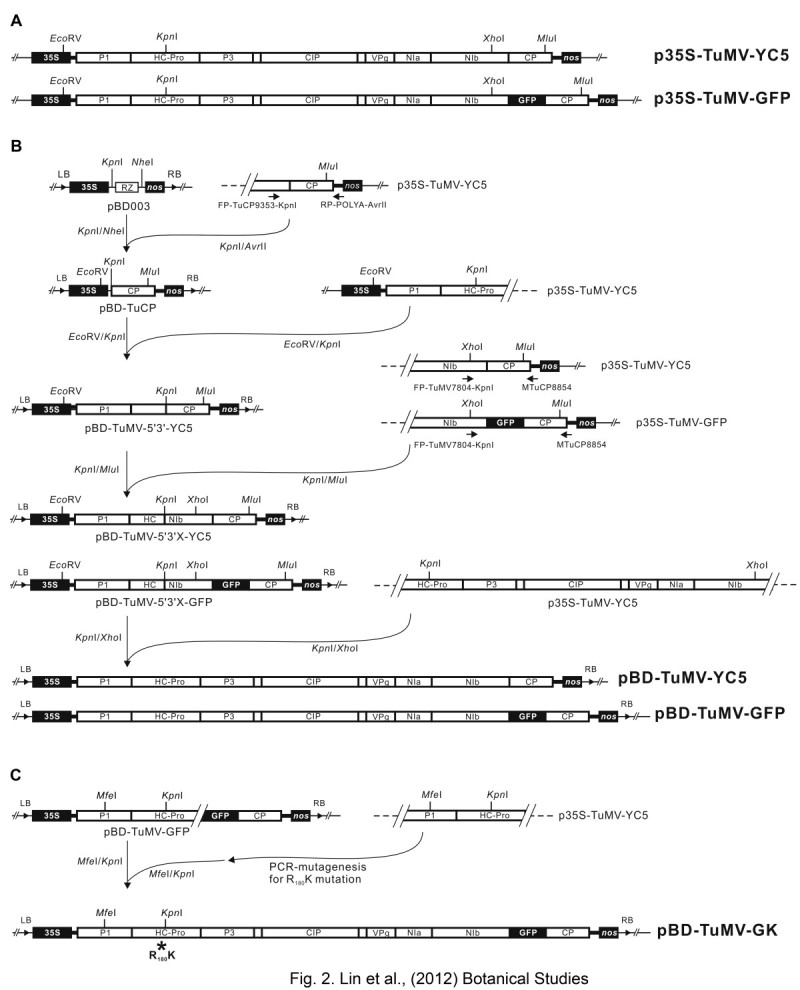
**Schematic representation of constructing pBD-TuMV infectious clones. (A)** Two TuMV *in vivo* infectious clones, p35S-TuMV-YC5 and p35S-TuMV-GFP (Lin et al., 2009; Niu et al., 2006), constructed on the pCaMVCN vector (Pharmacia) were designed for mechanical inoculation on *C. quinoa* plants. The two clones were used in the current study to construct a series of infectious constructs using a binary vector for agro-infiltration. **(B)** The full-length cDNA constructs of TuMV were constructed in the pBD003 binary vector (pBD-TuMV-YC5 and pBD-TuMV-GFP) for agrobacterium infiltration on *N. benthamiana* plants. The construction steps for cloning the TuMV full-length cDNA into the pBD003 binary vector are shown. The full-length TuMV cDNAs released from the p35S-TuMV series were constructed downstream of the CaMV *35S* promoter (*35S*) by insertions in between suitable restriction enzyme sites of pBD003. The final constructs were designated pBD-TuMV-YC5 and pBD-TuMV-GFP, contained a complete cDNA copy of wild type TuMV and TuMV carrying *GFP*, respectively. The coding regions of the cDNA are indicated by an open box. The 5′ and 3′ non-coding regions of the cDNA are indicated by heavy lines. **(C)** R_182_K mutation on HC-Pro of the TuMV was conducted by PCR-mediated mutagenesis. The mutated fragment was digested with *MfeI*/*Kpn* I and replaced the corresponding region of pBD-TuMV-GFP to generate pBD-TuMV-GK. An asterisk indicated the R_182_K mutation position.

The strategy for constructing the pBD-TuMV serial *in vivo* infectious clones is summarized in Figure 2. Two *in vivo* TuMV infectious clones, p35S-TuMV-YC5 and p35S-TuMV-GFP, constructed on the pCaMVCN vector were provided by Dr. Shyi-Dong Yeh (Figure 2A) (Lin et al., 2009; Niu et al., 2006). The 3′-end fragment of the TuMV was amplified by PCR with the primers FP-TuCP9353-KpnI (5′-AACCGGTACC GACCATACATGCCACGATATGGTCTTC-3′) and RP-POLYA-AvrII (5′-AGGTCGACGCGGCCGCCTAGG TTTTTTTTTTTTTTT-3′), which contain *Kpn* I, and *Avr* II (underlined), respectively. The PCR fragment was digested with *Kpn* I/*Avr* II and then ligated to the *Kpn* I/*Nhe* I-digested pBD003 vector to generate pBD-TuCP. Next, the *Eco* RV/*Kpn* I-digested fragment from p35S-TuMV-YC5 was ligated into the *Eco* RV/*Kpn* I-digested pBD-TuCP vector to generate pBD-TuMV5′3′-YC5. The primers FP-TuMV7804-KpnI (5′- GGAAAGGTACC CGTGGATGATTTCAACAAC -3′) containing *Kpn* I (underlined), and MTuCP8854 (5′- GCCTCTCTCGTTCCTTTTCT -3′) were used to amplify the region between the *NIb* and *CP* genes from p35S-TuMV-YC5 and p35S-TuMV-GFP, respectively. The *Kpn* I/*Mlu* I-digested fragment from p35S-TuMV-YC5 was ligated with the same restriction enzyme-digested pBD-TuMV-5′3′-YC5, to generate pBD-TuMV5′3′X-YC5. In addition, the *Kpn* I/*Mlu* I-digested fragment from p35S-TuMV-GFP was ligated with *Kpn* I/*Mlu* I-digested pBD-TuMV-5′3′-YC5 to generate pBD-TuMV-5′3′X-GFP. Finally, the fragment that was digested by *Kpn* I/*Xho* I from p35S-TuMV-YC5 was ligated with the same enzyme-digested pBD-TuMV-5′3′X-YC5 and pBD-TuMV-5′3′X-GFP to generate pBD-TuMV-YC5 and pBD-TuMV-GFP, respectively (Figure 2B).

For the mild strain of TuMV construction, the R_182_K mutation was introduced on the *HC-Pro* gene by PCR mutagenesis on p35S-TuMV-YC5, following the method demonstrated by Lin et al. (2009). The HC-Pro^R182K^ sequence was digested with *Mfe* I/*Kpn* I and ligated with the same enzyme-digested pBD-TuMV-GFP to generate pBD-TuMV-GK (Figure 2C).

### Infectivity assay of *in vivo* full-length cDNA clones

Local lesion host *C. quinoa* and systemic host *N. benthamiana* were used for the *in vivo* infectious clone infectivity assay. An aliquot of 10 μl (1 μg/μl) of individual constructs were mechanically introduced to leaves of *C. quinoa* with carborundum-dust, and the development of local lesions was recorded at 7 days post-inoculation (dpi). The mock (control) was mechanically inoculated with 10 μl ddH_2_O. For the agrobacterium mediated transformation activity assay, the *Agrobacterium tumefaciens* strain C58C1, which carries pBD-TuMV serial *in vivo* infectious clones, was incubated in an LB medium supplemented with kanamycin (100 μg/μl) at 28°C for 16 h. Thereafter, agro-infiltration was applied following the standard protocol (Llave et al., 2000). The symptoms and GFP fluorescence were observed after 6 days of infiltration.

### Purification of recombinant TuMV CP and production of antisera

The TuMV *coat protein* (*CP*) gene was amplified from the p35S-TuMV-YC5 infectious clone with primers PTu-CP-NdeI (5′- GTGTTTATCATATG GCAGGTGAGACG -3′) and MTu-CP-XhoI (5′- CAACTTCACTCGAG CTATAACCCCTTAACGC -3′), which contain *Nde* I, and *Xho* I (underlined), respectively. The PCR fragment was digested with *Nde* I/*Xho* I and ligated with the same restriction enzyme-digested pET-28b to generate pET-TuCP. The pET-TuCP was transferred into *E. coli* BL21 (DE3), which was treated with 0.5 M IPTG in LB medium for recombinant his-TuCP production. The recombinant his-TuCP was purified with Ni-NTA column by fast protein liquid chromatography (FPLC) (AKTApurifier, GE Healthcare) and used for antisera production that were produced from New Zealand white rabbits, as described by Lin et al. (2002). The titer of TuMV CP antisera was analyzed by Enzyme-linked immunosorbent assay (ELISA) and western blotting.

### Enzyme-linked immunosorbent assay (ELISA)

For verification of virus infection, each sample represented 3 inoculated leaves and 3 systemic leaves collected from each of the 3 repeated plants, with 3 leaf-discs (0.6 cm in diameter) punched from each leaf. These collected samples were assayed by indirect ELISA using the polyclonal antiserum to the TuMV CP. Goat anti-rabbit immunoglobulin G conjugated with alkaline phosphatase (Amersham Biosciences) was used as the secondary antibody, and *p*-nitrophenyl phosphate (Sigma) was used as the substrate for color development. Results were recorded by measuring absorbance at 405 nm (which were stained for 5 min after the addition of the substrate) using an ELISA reader (Perkin Elmer).

The analysis of variance (ANOVA) was performed at first to test the levels of absorbance under different conditions. It was followed by the pairwise comparison between two conditions of interest using Tukey’s honest significant difference (HSD) test.

### Western blot analysis

The systemic leaves from inoculated plants were homogenized in 20 volumes (wt/vol) of denaturing buffer (50 mM Tris–HCl, pH 6.8, 4% SDS, 2% 2-mercaptoethanol, 10% glycerol, and 0.001% bromophenol blue). After incubation at 100°C for 5 min, extracts were clarified by centrifugation at 8,000 *g* for 3 min. Total proteins were separated by SDS gel electrophoresis, and western blots were analyzed using the antiserum to TuMV CP. Gels were stained with Coomassie brilliant blue R250, and levels of the large subunit of RUBISCO (molecular mass, 55 kDa) were used as loading controls.

## Results

### Construction of a mini binary vector – pBD003

The 4 kb mini binary vector, pBD003, contains LB and RB sequences. Between the LB and the RB sequences, the construct contains a CaMV *35S* promoter, a *kanamycin* resistance gene (*Kan*^*R*^), *pSa* origin, *E. coli* replication origin (*Ori*), *nos* terminator, and ribozyme site (*RZ*) (Figure 1). The role of *nos* terminator is to help generate a polyA tail for the polyA-type viruses, such as potyvirus, whereas the ribozyme site is designed for the non-polyA viruses. In addition, commonly used restriction enzyme sites were removed from the pBD003 backbone, which reduces the problems of introducing inserts for cloning purposes (Figure 1).

#### Construction of full-length cDNA clones with *35S* promoter in the mini binary vector

The construction of three pBD-TuMV clones was based on two plasmids, p35S-TuMV-YC5, and p35S-TuMV-GFP. The p35S-TuMV series all contain full-length TuMV cDNA between the CaMV *35S* promoter and *nos* terminator (Figure 2A) in which the ribozyme sequence was removed during this construction. The p35S-TuMV-GFP contains the *GFP* gene between *NIb* and *CP* genes of the TuMV (Figure 2A).

The pBD-TuMV series contained the full-length cDNA sequence of TuMV-YC5 downstream from the *35S* promoter and located between LB and RB based on the binary vector pBD003. The scheme for the construction of TuMV full-length clones in the binary vector pBD003 from the p35S-TuMV series is shown in Figure 2B. Moreover, the R_182_K mutant of HC-Pro was created on pBD-TuMV-GFP clone by PCR-mediated mutagenesis to generate pBD-TuMV-GK (Figure 2C). The pBD-TuMV-GK was used for further investigation in this study.

#### Initial infectivity assay of *in vivo* full-length cDNA clones on *C. quinoa* plant

**Figure 3:**
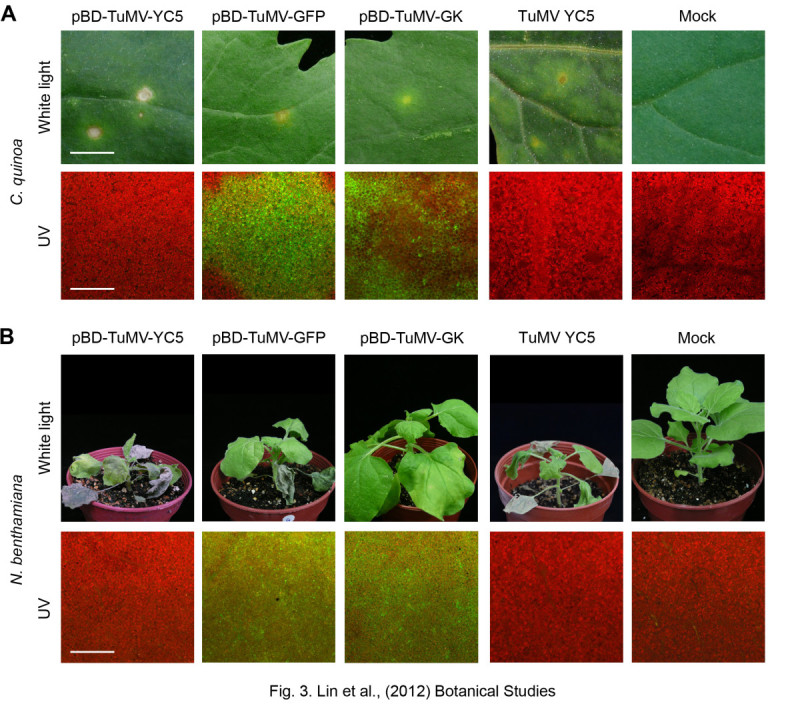
**Infectivity assay of pBD-TuMV-YC5, pBD-TuMV-GFP and pBD-TuMV-GK. (A)** The 3 constructs were directly mechanically introduced to *C. quinoa* plants, and local lesions appeared on the inoculated leaves at 7 dpi (upper panel). The virus culture of TuMV YC5 and Mock-inoculation were used as positive and negative controls, respectively. Bar, 0.5 cm. The GFP fluorescence expressed by pBD-TuMV-GFP and pBD-TuMV-GK is shown in the lower panel. Bar, 25 μM. **(B)** *N. benthamiana* plants were infiltrated with agrobacterium individually carrying pBD-TuMV-YC5, pBD-TuMV-GFP, and pBD-TuMV-GK. The virus culture of TuMV YC5 and Mock-inoculation were used as positive and negative controls, respectively. The photographs were taken at 14 dpi (upper panel). GFP expression was detected with a fluorescent microscope (lower panel).

To test the infectivity of the pBD-TuMV serial clones, *C. quinoa* plants were mechanically inoculated with individual pBD-TuMV plasmids. Local lesions appeared after 7 dpi, whereas the mock did not show any local lesions (Figure 3A). Moreover, green fluorescence appeared on the leaves inoculated with pBD-TuMV-GFP and pBD-TuMV-GK, whereas no green fluorescence appeared on the pBD-TuMV-YC5 or mock-inoculated leaves (Figure 3A, lower panel). In addition, the virus culture of TuMV YC5 was also mechanically inoculated on *C. quinoa* plant as a positive control (Figure 3A); the lesion morphology and developing time was identical to the inoculation of the pBD-TuMV-YC5 plasmid. These results indicated that the 3 pBD-TuMV plasmids were infectious, and that pBD-TuMV-GFP and pBD-TuMV-GK expressed the *GFP* gene.

The TuMV mild strain (TuMV-GK) with R_182_K mutation on the *HC-Pro* gene showed mild symptoms on *N. benthamiana* plants (Figure 3B). Chlorotic spots appeared on TuMV-GK-infected *C. quinoa* without necrosis (Figure 3A). In addition, the TuMV-GK infected tissue showed weaker green fluorescence in both plants compared with those produced by WT TuMV (Figure 3). Moreover, the WT TuMV produced green fluorescence spots on *C. quinoa*, but TuMV-GK produced a green fluorescence ring on *C. quinoa* (Figure 3A).

#### Transformation analysis by agro-infiltration

The biological activity of the pBD003 binary vector for the agrobacterium-mediated transformation was tested, and the infectivity of the pBD-TuMV series was evaluated on the TuMV systemic host *N. benthamiana* by agro-infiltration, whereas the virus culture of TuMV YC5 was mechanically inoculated on *N. benthamiana* plant as a positive control. The pBD-TuMV-GFP successfully infected the systemic host *N. benthamiana* by agro-infiltration, with infected plants showing mosaic, necrotic spots, leaf curling and wilting symptoms (Figure 3B, upper panel). Fluorescent microscopy revealed that pBD-TuMV-GFP agro-infiltrated plants expressed green fluorescence on their leaves, whereas pBD-TuMV-YC5, the virus culture of TuMV YC5, and healthy plants showed the original red fluorescence of chloroplasts (Figure 3B, lower panel). Moreover, pBD-TuMV-GK showed mild symptoms on *N. benthamiana* plants, namely mild curling of new leaves, but these symptoms recovered after a few days (Figure 3B). The results indicated that the pBD-TuMV series are infectious by agro-infiltration.

**Figure 4:**
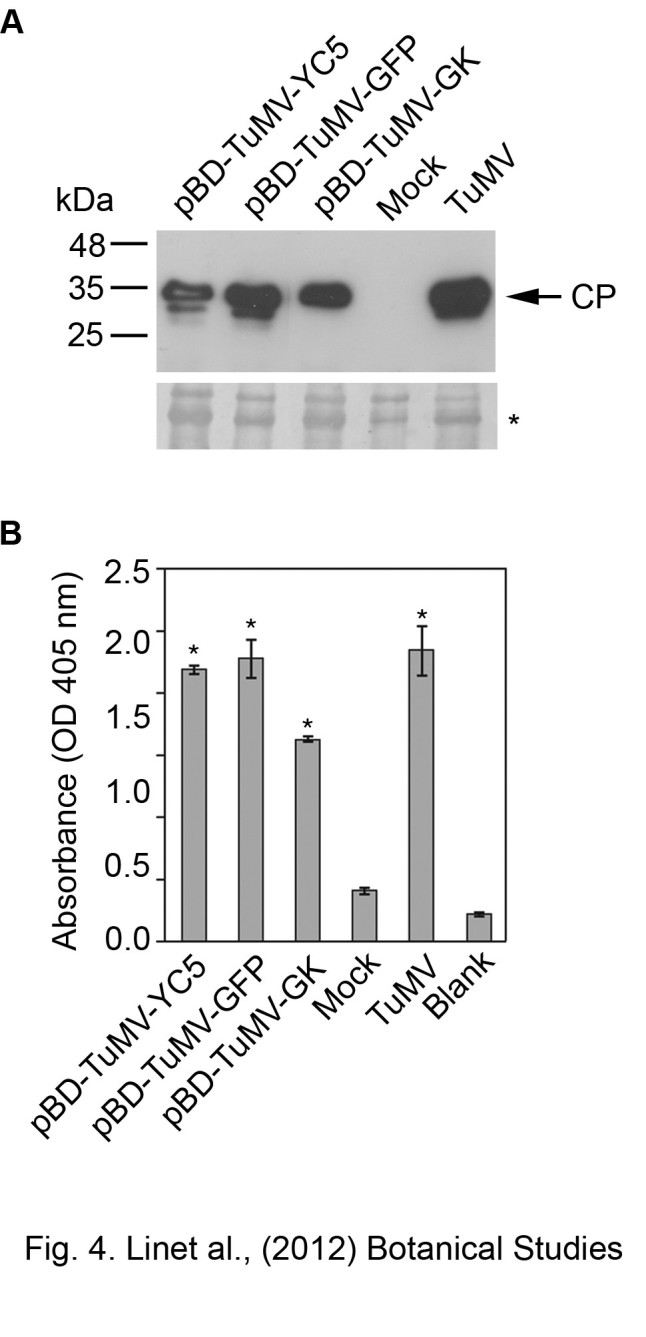
**Validation of the infectivity of pBD-TuMV-CY5, pBD-TuMV-GFP and pBD-TuMV-GK on** ***N. benthamiana*** **plants.** The agro-infiltrated *N. benthaminana* plants were analyzed with western blotting **(A)** or indirect ELISA **(B)** at 7 dpi, using the antiserum to TuMV CP. The samples of agro-infiltrated leaves are individually indicated. The sample from leaves infected by TuMV virus culture (14 dpi) that was used as positive control is indicated as TuMV. The mock was inoculation was used as a negative control. The blank indicates the wells coated with buffer for basal-level control. The TuMV CP antiserum was used at 10,000x dilution for both assays. The samples significantly different with mock (*p*-value < 0.05) were indicated as “*”. Bars represent standard deviations (*n* = 3).

All inoculated plants were analyzed by western blot and indirect ELISA to confirm the infection. The *N. benthamiana* plants with severe symptoms reacted strongly to TuMV CP antiserum, and the healthy control showed negative reactions (Figure 4A & B). By western blotting analysis, a 35 kDa protein corresponding to the CP of TuMV was detected in plants agro-infiltrated by the TuMV *in vivo* infectious clones (Figure 4A). The results also indicated that the antisera to TuMV CP produced by recombinant protein can specifically detect TuMV infection as analyzed by ELISA or western blot (Figure 4).

#### Evaluation of the initial infectivity efficiency of the pBD-TuMV-series infectious clones

To test whether the pBD-TuMV-series infectious clones could improve initial infection efficiency, we compared the success infection rates of the *N. benthamiana* plant by agro-infiltration versus the mechanical inoculation. Here, the mechanical inoculation was used 4 local lesions of *C. quinoa* tissue as inoculums. In addition, the severe strain (TuMV-GFP) and the mild strain (TuMV-GK) were evaluated, as shown in Table 1. The success rate for pBD-TuMV-GFP initial infection on *N. benthamiana* was 100% by agro-infiltration, whereas the success rate was only 10% by mechanical inoculation when the inoculums were taken from 4 local lesions of *C. quinoa* (Table 1). The results indicate a low success rate for TuMV initial infection transferred from *C. quinoa* plants to *N. benthamiana*; agro-infiltration eliminated this problem by directly generating the TuMV on *N. benthamiana*, which sped the initial infection. In addition, the data showed lower initial infectivity of TuMV-GK (10% success rate) using mechanical inoculation from local lesions of *C. quinoa* to *N. benthamiana* (Table 1). However, TuMV-GK showed a 53.3% initial infection rate on agro-infiltrated *N. benthamiana* (Table 1). This finding indicates that agro-infiltration can also eliminate the problem of low infectivity of the TuMV that has HC-Pro mutation.

**Table 1:** **Comparison of initial infectivity for agro-infiltration versus mechanical inoculation**

Virus	Inoculation method	Systemic leaf	Initial infectivity (%)^c^
TuMV-GFP	A.I.^a^	10/10	100
	M.I.^b^	1/10	10
TuMV-GK	A.I.	8/15	53.3
	M.I.	1/10	10

^a^A.I., the inoculation method was applied by agro-infiltration with pBD-TuMV-GFP or pBD-TuMV-GK on *N. benthamiana* plants. ^b^M.I., individual lesions on *C. quinoa* plants infected with pBD-TuMV-GFP or pBD-TuMV-GK were collected and used as inocula for mechanical inoculation. ^c^Number of infected plants as a percentage of the total plant population. The virus infection was analyzed by ELISA at 12 dpi.

#### The pBD003 produced the bursts of transferred RNA to advance the viral RNA accumulation

**Figure 5:**
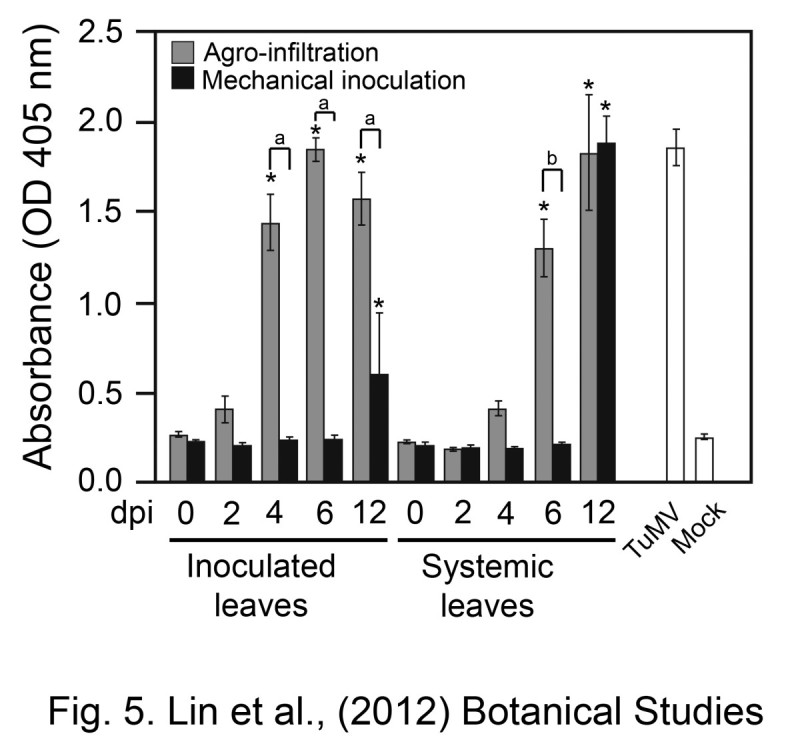
**Comparison of TuMV initial infection efficiency between the agro-infiltration (pBD-TuMV-YC5) and mechanical inoculation (virus culture of TuMV YC5).** Virus accumulated *in vivo* on inoculated or systemic leaves of *N. benthamiana* plants in which infection was initiated by either agro-infiltration or mechanical inoculation with TuMV infectious clones. The numbers indicated the days after inoculation (dpi). The virus titer was detected by ELISA with TuMV CP antiserum. The 12 dpi of TuMV-infected *N. benthamiana* plants (TuMV) were used as a positive control, whereas the mock-inoculated plants (Mock) were the negative control. The samples of 2, 4, 6, and 12 dpi significantly different with the sample of 0 dpi in the same inoculation method and leaves position (*p*-value < 0.05) are indicated as “*”. Significant difference between the agro-infiltration and the mechanical inoculation of the inoculated leaves under the same dpi (*p*-value < 0.05) is indicated as “a”. Significant difference between the agro-infiltration and the mechanical inoculation of the systemic leaves under the same dpi (*p*-value < 0.05) is indicated as “b”. Bars represent standard deviations (*n* = 3).

We compared the TuMV accumulated rates for mechanical inoculation with virus culture of TuMV YC5 versus agro-infiltration with pBD-TuMV-YC5 by ELISA (Figure 5). Here, the mechanical inoculation was used TuMV YC5-infected *N. benthamiana* tissue as inoculuns. The *N. benthamiana* plants infected with virus culture of TuMV YC5 by mechanical inoculation, show the leaf-curing symptoms on the systemic leaves at 12 days after mechanical inoculation; the ELISA readings of TuMV CP (OD_405_) showed TuMV-positive signals on the inoculated (OD_405_ 0.6 ± 0.34) and systemic leaves (OD_405_ 1.88 ± 0.14) (Figure 5). No significance symptoms were observed on either the inoculated leaves or systemic leaves between 0 to 6 dpi. The ELISA readings (OD_405_) averaged 0.22 ± 0.02 at 0 to 6 days after mechanical inoculation in inoculated and systemic leaves (Figure 5). TuMV infection was not detectable during the time less than 6 dpi.

Using the agro-infiltration method with pBD-TuMV-YC5, the inoculated leaves showed that the readings of OD_405_ were 0.4 ± 0.07 (2 dpi), 1.44 ± 0.15 (4 dpi), and 1.85 ± 0.06 (6 dpi) and the systemic leaves showed that the readings of OD_405_ were 0.41 ± 0.04 (4 dpi) and 1.3 ± 0.16 (6 dpi) (Figure 5). Our results indicated that the agro-infiltration method results in bursts of TuMV viral RNA in inoculated leaves by the expression of pBD-TuMV-GFP resided in T-DNA, and these abounding viral RNAs enhance the TuMV systemic infection efficiency.

## Discussion

A commercialized mini binary vector (pGreen) is available (Hellens et al., 2000). However, some restriction sites existing on the pGreen backbone might interfere with further cloning procedures of the TuMV infectious clone. Therefore, we designed a mini binary vector that is specifically suitable for prompt construction of TuMV infectious clones. In this study, a mini binary vector (pBD003) for agrobacterium-mediated transient expression was created. The vector was then applied to deliver virus infectious clones to the nuclei of plant cells. The vector was 4,141 bp in size and unnecessary restriction sites were removed from the plasmid backbone. We used the strategy of compatible cohesive end of restriction enzymes, such as *Avr* II ligated with *Nhe* I, to link various comportments to avoid restriction sites remaining on the vector after ligation. The advantage of a plasmid with fewer restriction sites on the vector backbone can increase the usage of restriction sites. Moreover, having a miniature-sized vector can increase the rate of successful construction. This is especially useful when handling the large fragments for recombination analyses, genomic mutation, or inserting a foreign open-reading frame into the infectious clone. The pBD003 contains a ribozyme to cleave the nascent RNA at the 3′-end for generation of infectious clones for non-poly A tail type of viruses. In addition, pBD003 also has a *nos* terminator, which provides a polyadenylation signal to generate a poly A tail on viral RNA. In our case of TuMV, the ribozyme sequence has been removed in the plasmid of pBD-TuMV series.

In pBD003, the *Kpn* I and *Nhe* I sites on the vector can be used for other viral infectious clone constructions. In the future, the more restriction enzyme sites will be introduced to the pBD003 for cloning convenience. However, these restriction sites immediately after the *35S* promoter must be removed by site-directed mutagenesis because the non-viral sequence between the *35S* promoter and 5′-end in viral RNA affects the initial infectivity (Lin et al., 2002; Maiss et al., 1992; Shi et al., 1997).

The plasmids of serial pBD-TuMV infectious clones can be applied directly on *C. quinoa* by mechanical inoculation to induce local lesions, indicating that these pBD-TuMV constructs possess viral infectivity. Moreover, the pBD-TuMV series caused systemic infection on *N. benthamiana* by agro-infiltration, which indicated that the pBD-TuMV series can be transferred through an agrobacterium transformation mechanism. In addition, agrobacterium-mediated inoculation overcame the low infectivity rate when transferring the inocula prepared from *C. quinoa* plants to *N. benthamiana* plants. The reason for the low infectivity might due to the presence of components from *C. quinoa* plants that affect TuMV infection in *N. benthamiana* plants. However, this phenomenon did not show on ZYMV infection (Lin et al., 2007), suggesting that various viruses might have varying susceptibility to the components of *C. quinoa* during infection.

Virus accumulation was detected after 10 dpi in plants inoculated by the virus culture of TuMV CY5, whereas by agro-infiltration, pBD-TuMV-CY5 caused viral accumulation on inoculated leaves at 4 dpi (Figure 5). Our explanation for this phenomenon is that agrobacterium was highly effective in transferring T-DNA of the infectious clone into the nucleus, and these T-DNAs constitutively expressed TuMV viral RNA. We cannot exclude the possibility of synergistic symptoms caused by agrobacterium on primary infiltration of the *N. benthamiana* plants. However, this concern can be eliminated by the secondary transfer of TuMV from the primary infiltrated *N. benthamiana* plants to new plants. The secondarily inoculated plant will not have the interfering from agrobacterium. Therefore, the efficiency of transient expression of agro-infiltration plays an important role on initial infectivity. The 53% initial infectivity of pBD-TuMV-GK might be caused by the variation in efficiency of the transient expression of agro-infiltration or might be because of the less infectious nature of the clone (Table 1).

Because of the highly effective transient expression of pBD003, the initial infectivity rate also increased in the TuMV-GK mild strain through pBD003-delivered inoculation (Table 1). The pBD003-delivered TuMV-GK demonstrated 53.3% initial infectivity, and also shortened the process of infectivity, compared with the regular inoculation process using the p35S-TuMV infectious clone. The HC-Pro mutant (R_182_K) was responsible for the low efficiency of the mild strain’s initial infectivity, which consequently affected viral replication and resulted in low amounts of viral genome RNA. The TuMV-GK and ZYMV mild strain (ZYMV GAC) have a common R_180_ mutation on HC-Pro, which showed mild symptoms and a green fluorescence ring on *C. quinoa* (Figure 3A) (Lin et al., 2007). The results indicating the highly conserved R_180_ of HC-Pro of the potyvirus is a critical amino acid for symptoms development and local lesion formation of *C. quinoa*. Our previously study also showed that R_180_ of HC-Pro also plays an important role on suppression of the microRNA-mediated gene silencing pathway (Wu et al., 2010). In addition, the TuMV-GK also provides a good cross-protection on *N. benthamiana* to against the wild type TuMV (data not shown), indicating that the TuMV-GK can be used as a model for studying the mechanism of cross-protection in the future.

## Conclusion

The newly constructed mini binary vector pBD003 provides a useful tool for research into host-virus interactions, viral infectious clone infectivity construction, and transient expression. The backbone of pBD003 contains no commonly used restriction enzyme sites, and the miniature size of the vector is advantageous for cloning. In this study, we provided a high initial infectivity of viral vector to study the virology and the functional genome of the virus. It can also be used to screen more TuMV mild strains that have various mutations on the viral genome for the cross-protection. Further investigation of the pBD003 mini binary vector can also be used on transient expression for gene expression and cell localization through agrobacterium transformation.

## Authors’ original submitted files for images

Below are the links to the authors’ original submitted files for images. Authors’ original file for figure 1 Authors’ original file for figure 2 Authors’ original file for figure 3 Authors’ original file for figure 4 Authors’ original file for figure 5
