# Supplementary figures and images for: Improving initial infectivity of the Turnip mosaic virus (TuMV) infectious clone by an mini binary vector via agro-infiltration
Source: Bot Stud. 2013 Aug 28;54:22. doi: 10.1186/1999-3110-54-22 (PMC5432745; doi:10.1186/1999-3110-54-22)

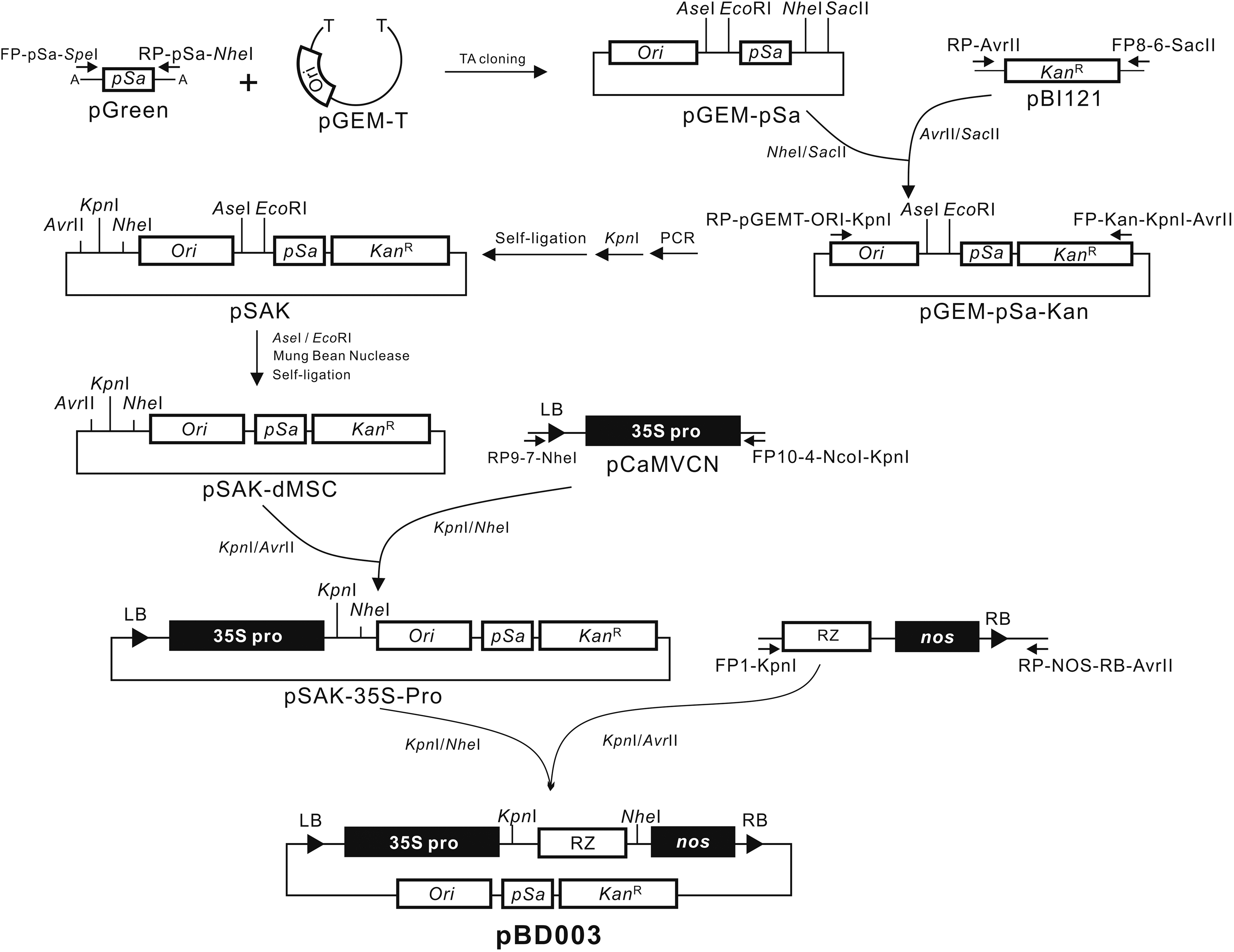

Supplement: Supplementary file 1 — Authors’ original file for figure 1 [file 40529_2012_29_MOESM1_ESM.tif]

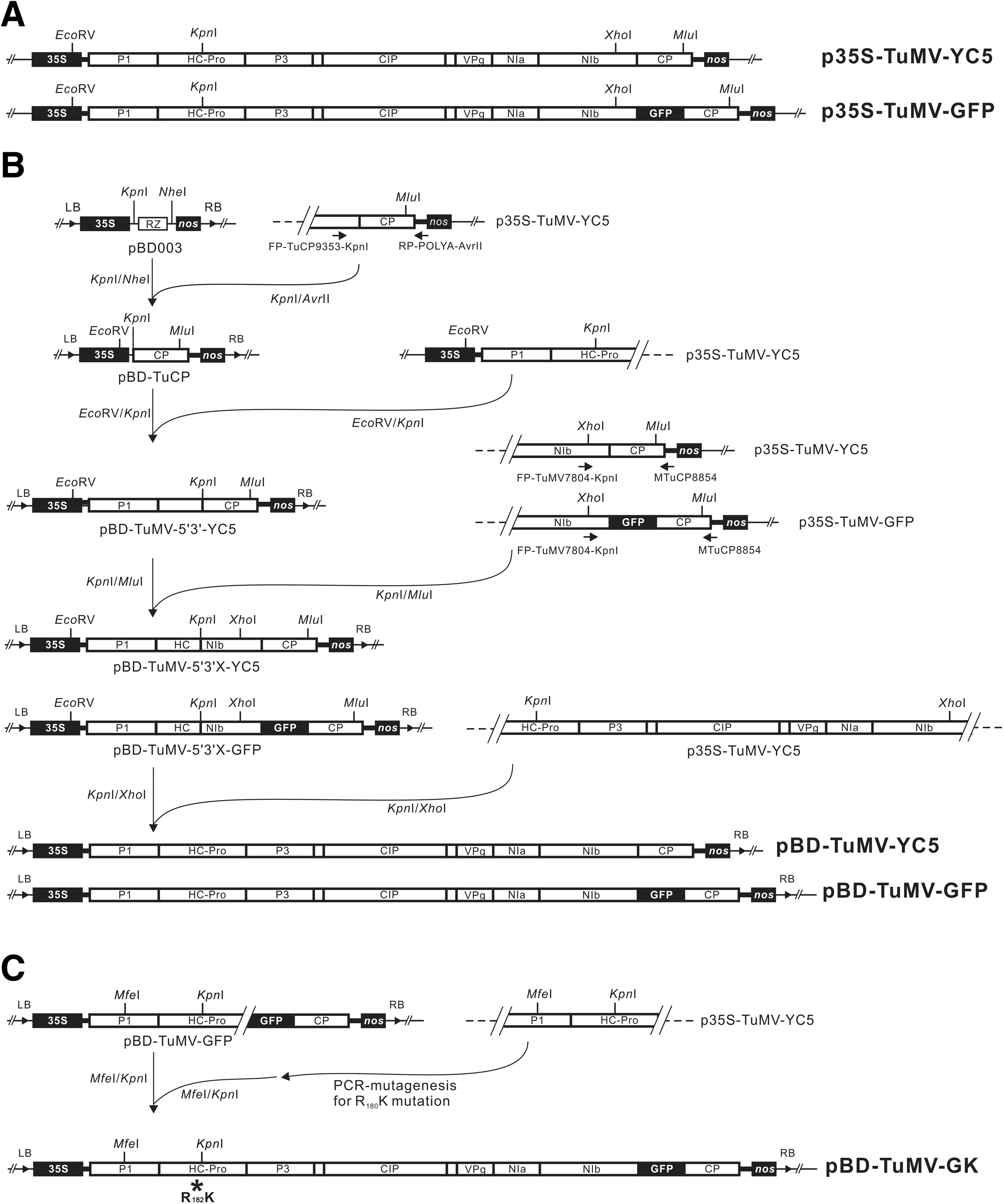

Supplement: Supplementary file 2 — Authors’ original file for figure 2 [file 40529_2012_29_MOESM2_ESM.tiff]

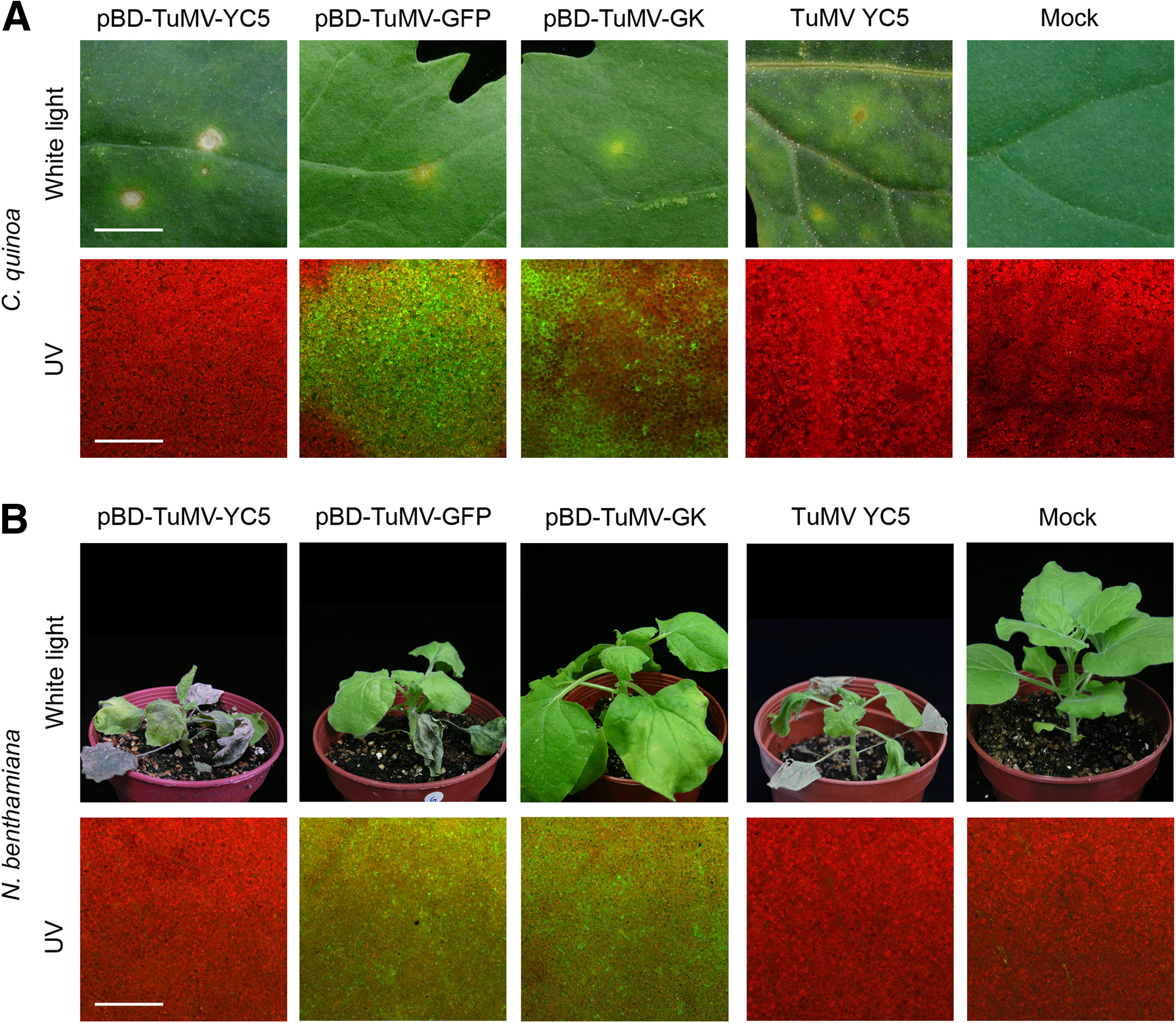

Supplement: Supplementary file 3 — Authors’ original file for figure 3 [file 40529_2012_29_MOESM3_ESM.tif]

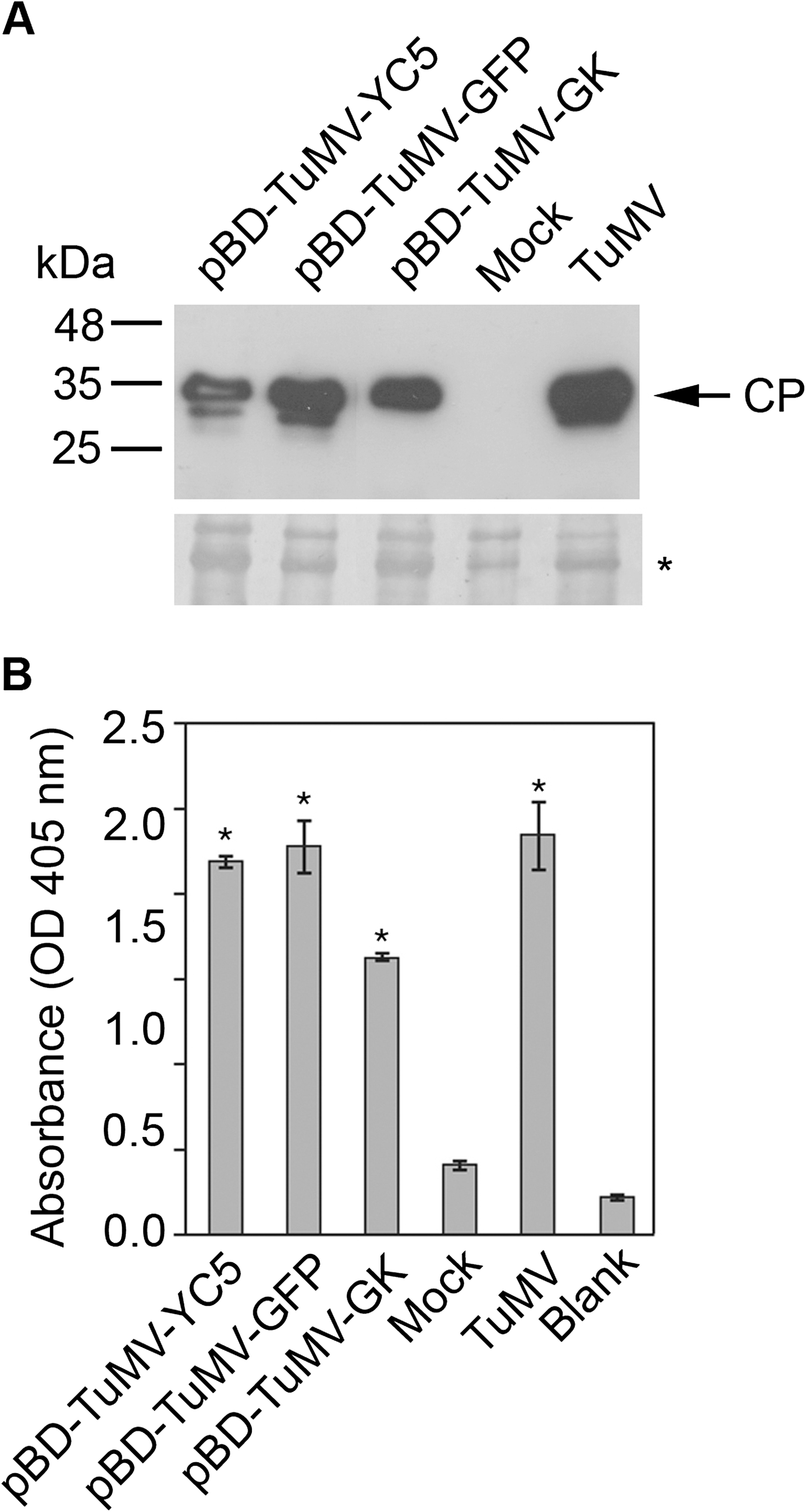

Supplement: Supplementary file 4 — Authors’ original file for figure 4 [file 40529_2012_29_MOESM4_ESM.tif]

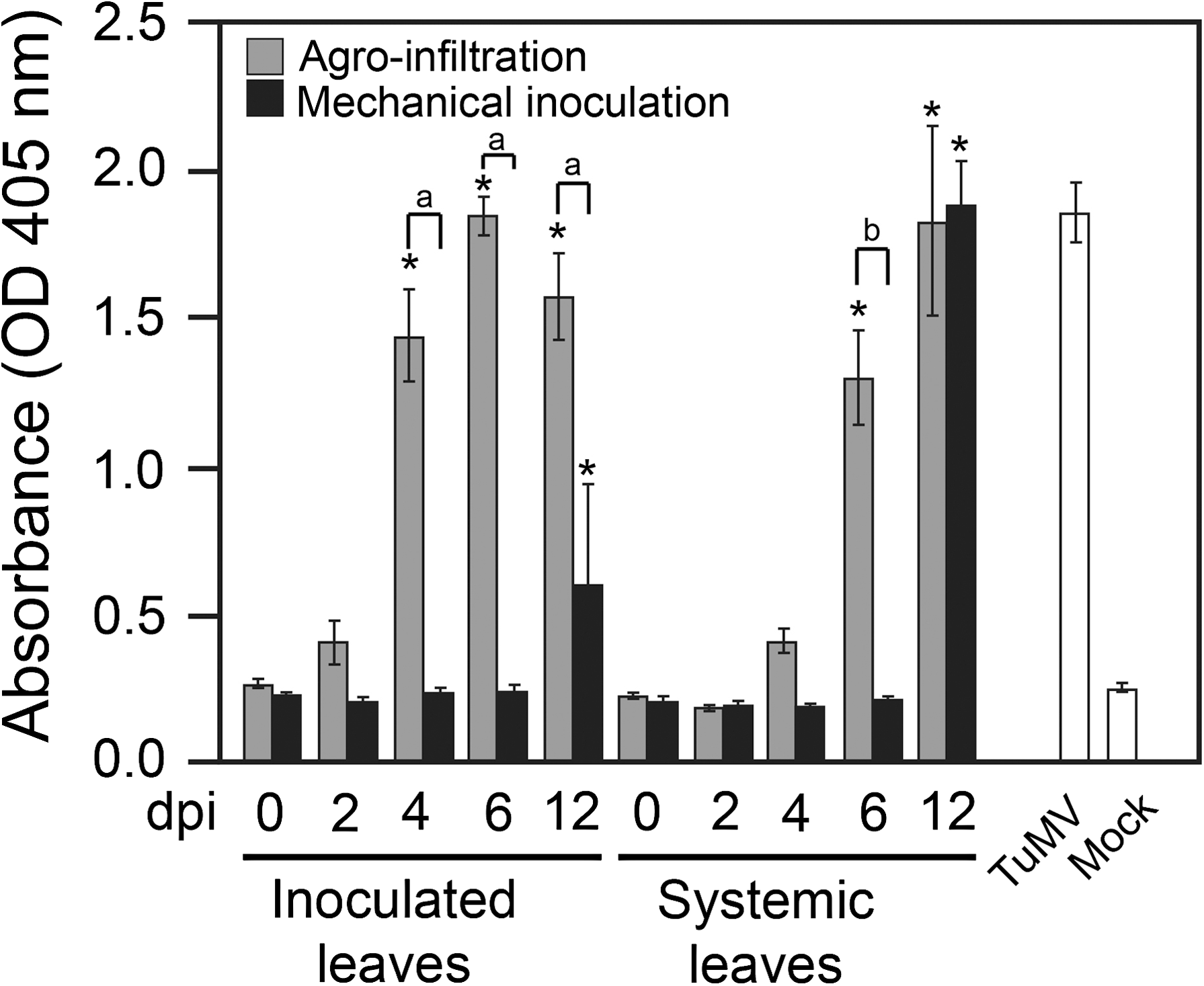

Supplement: Supplementary file 5 — Authors’ original file for figure 5 [file 40529_2012_29_MOESM5_ESM.tif]
